# Intelligent data governance and quality control for chest Bi syndrome/coronary heart disease across the prevention–treatment–rehabilitation continuum: integrating a standardized framework, adversarially-optimized trigger engine, and domain-adaptive AI

**DOI:** 10.3389/fmedt.2026.1759773

**Published:** 2026-08-12

**Authors:** Huisi Hong, Guiyuan Yang, Weihan Shen, Shiqi Luo, Yiming Yuan, Yong Zhao, Hong Zhang, Shuangyan Li, Juhua Wu, Wenge Chen, Shaoyang Men, Kaixuan Lin

**Affiliations:** 1 The Tenth Clinical Medical College, Guangzhou University of Chinese Medicine, Zhongshan, Guangdong, China; 2 Guangzhou University of Chinese Medicine, Guangzhou, Guangdong, China; 3 Guangdong University of Technology, Guangzhou, Guangdong, China

**Keywords:** adversarial learning, bilinear attention, chest bi syndrome (coronary heart disease), clinical data quality control, data governance, domain-adaptive AI, multi-tier trigger engine, TCM-advantaged diseases

## Abstract

**Background:**

Digital transformation in Traditional Chinese Medicine (TCM) is hindered by data silos, poor standardization, and inefficient quality control. This study developed and validated an intelligent data governance system to address these gaps.

**Methods:**

Using Chest Bi syndrome (coronary heart disease) as a model, we established a standardized data system (9 subsets, 352 elements; 42.6% TCM-specific) based on the “Triple-Loop Coupling” theory and HL7 FHIR (Fast Healthcare Interoperability Resources)/SNOMED CT standards. The system integrates a three-tier trigger engine (128 rules) for real-time alerts and a domain-adapted LLM, fine-tuned on TCM texts and guidelines, for unstructured data processing. Efficacy was evaluated via a prospective controlled study (*n* = 972).

**Conclusion:**

The intervention group showed significant improvements (*P* < 0.001): data completeness rose from 82.51% to 99.59% (*Δ*=17.08%), and TCM-specific data elements collection improved from 67.08% to 98.97% (*Δ*=31.89%). Logical consistency errors dropped from 15.84% to 1.65% (*Δ*=-14.20%). Unstructured data conversion increased from 45.06% to 91.77% (*Δ*=46.71%). Quality control time per record decreased by 58.95% (from 12.48 to 5.12 min). The AI model achieved a 91.4% F1-score and 91.8% alert accuracy.

**Conclusion:**

The “standardization-trigger-AI” model significantly enhances data quality and efficiency for TCM. This system provides a preliminary and verifiable exploratory path for the intelligent governance of TCM clinical data, offering a scalable solution for TCM modernization.

## 1 Introduction

TCM consistently applies the core philosophy of “emphasizing primary prevention (preventing onset), secondary prevention (halting progression), and tertiary prevention (averting recurrence)” across the entire continuum of prevention, treatment, and rehabilitation for cardiovascular and other chronic conditions where it demonstrates distinct therapeutic advantages (1, 2). Its diagnostic and therapeutic value is widely recognized in clinical practice (3).

As a representative condition within the TCM cardiovascular domain, Chest Bi syndrome (coronary heart disease) requires the integration of multi-source data—encompassing pre-diagnosis risk assessment, syndrome differentiation and treatment during diagnosis, and post-treatment rehabilitation follow-up—to accurately delineate the dynamic patterns of syndrome evolution (4–6). However, current clinical data management in TCM faces three major challenges: First, significant data fragmentation exists due to the absence of standardized interfaces for integrating multi-source heterogeneous data, such as electronic medical records (EMR), tongue and pulse imaging, laboratory findings, and follow-up records, which impedes seamless data flow (7, 8). Second, standardization remains inadequate, with TCM-specific data elements—including descriptions of the Four Diagnostic Methods, syndrome differentiation, and herbal formula compositions—often recorded in unstructured free text. This reliance on individual practitioner experience results in a lack of machine-readable specifications (2, 9). Third, quality control models are outdated. Traditional manual audits, typically conducted through *post-hoc* sampling, are not only labor-intensive and time-consuming but also susceptible to subjective errors, leading to oversights and inaccuracies. Consequently, data quality issues are frequently identified only during subsequent scientific analysis or efficacy evaluation stages (2, 9).

Advances in data governance and artificial intelligence are creating new pathways to overcome these challenges. In data standardization, HL7 FHIR—as an internationally recognized data exchange standard—has demonstrated significant advantages in cross-system data integration (10, 11). For automated quality control, rule-based trigger engines can identify data anomalies in real time; however, existing solutions are primarily designed for Western medical data and remain inadequately adapted to the distinctive characteristics of TCM diagnosis and treatment (11). In intelligent processing, while large language models such as BERT have shown strong performance in clinical natural language processing, those without domain-specific tuning often fail to accurately recognize TCM-specific entities and semantic relationships (11–13). Recent studies have highlighted the transformative role of AI-driven frameworks in enhancing data integration and semantic interoperability across heterogeneous healthcare systems (14).

Current research predominantly concentrates on isolated technologies or singular stages. For instance, Yang Fang et al. (18) investigated the theoretical value of integrated “prevention–treatment–rehabilitation” management but did not elaborate on specific technical implementations. Zhang Chunyan et al (19). applied artificial intelligence to auxiliary TCM diagnosis yet overlooked data quality control aspects. Luo Hao et al (20). developed a specialized cardiovascular disease database but inadequately standardized TCM-specific indicators. More critically, existing studies lack solutions that deeply integrate standardized data governance, real-time triggering mechanisms, and domain-adaptive AI. They also fail to effectively address persistent issues such as trigger false positives and multimodal data distribution discrepancies (15–20).

Building upon these advancements in AI-driven interoperability, there remains a need for an integrated system tailored to the unique logic of TCM clinical pathways. Inspired by the integrated “adversarial learning+attention mechanism” approach of the existing model (21, 22), this study developed an intelligent data governance and quality control system (23) using Chest Bi syndrome (coronary heart disease) as a demonstration disease. The research objectives were:

1. To establish a standardized data element system encompassing the entire clinical pathway while highlighting TCM-specific characteristics (24–26);
2. To design an adversarially optimized three-tier trigger engine—a self-learning filtering mechanism aimed at significantly reducing false positive alerts and physician alarm fatigue (27, 28);
3. To develop a domain-adaptive AI model enhanced with bilinear attention mechanisms for improved processing of TCM textual data (29–31);
4. To validate the system's effectiveness and practical utility through prospective controlled studies (32–34), thereby providing a preliminary and verifiable exploratory path for the digital transformation of TCM data governance.

## 2 Methods

### 2.1 Overall system architecture

**Figure 1:**
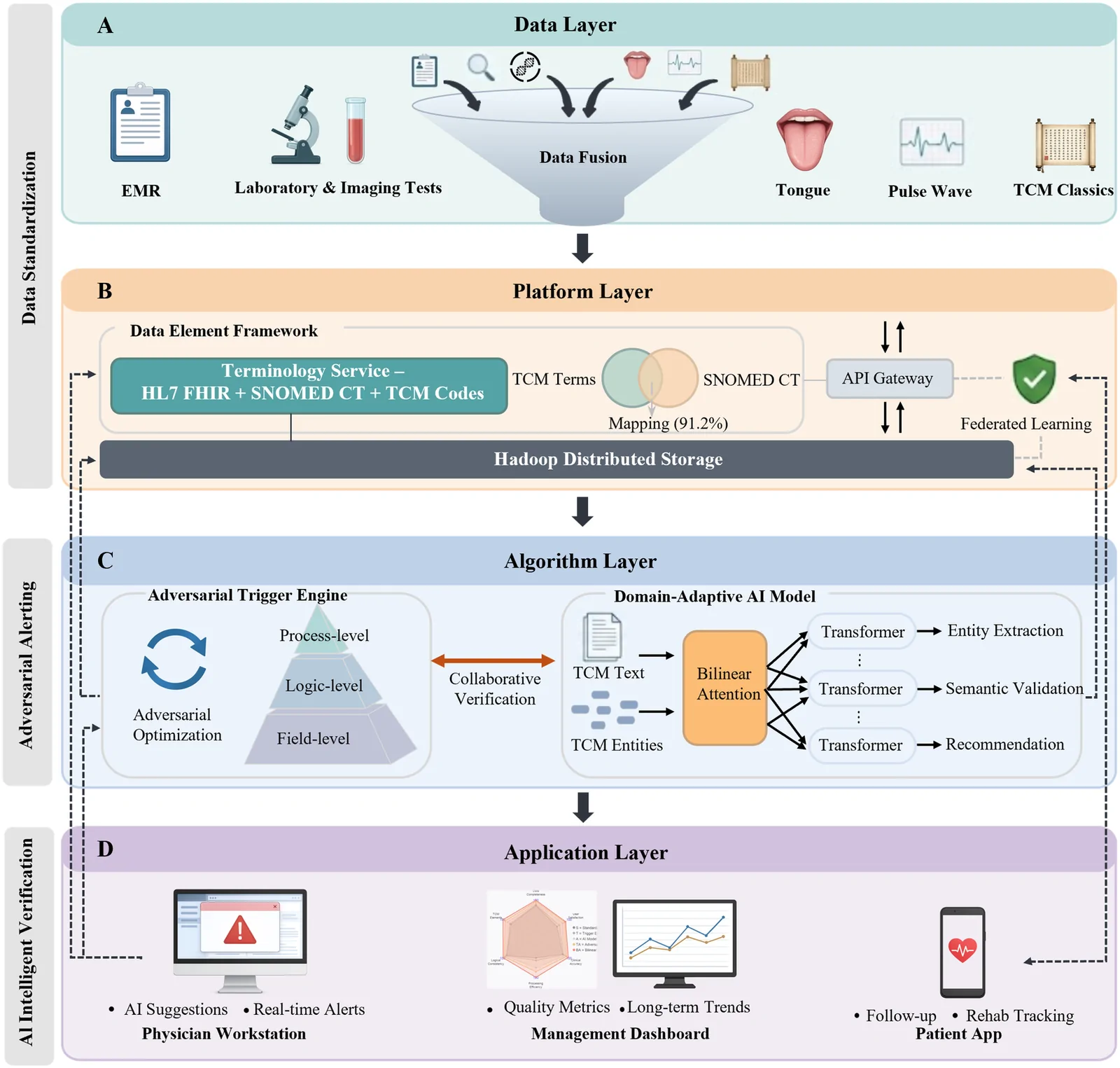
The architecture of the intelligent data governance and quality control system for chest impediment (Bì syndrome). Based on the "Three-ring Coupling" theory, the framework is structured into four layers: **(A)** Data Layer: Aggregates multi-source heterogeneous data, including EMRs, tongue/pulse images, and TCM classics. **(B)** Platform Layer (Standardization Ring): Establishes a standardized data element framework based on HL7 FHIR and SNOMED CT (achieving 91.2% mapping coverage), integrated with federated learning for privacy protection. **(C)** Algorithm Layer (Adversarial Trigger Ring): Features the synergy between an Adversarial Optimized Triple-level Trigger Engine (for real-time logic verification) and a Domain-Adaptive AI Model with Bilinear Attention (for semantic entity extraction and consistency checking). **(D)** Application Layer (Feedback Ring): Facilitates a closed-loop management system via the physician workstation, management dashboard, and patient app, supporting continuous model iteration through user feedback.

Guided by the “Triple-Loop Coupling” theory—defined here as a continuous closed-loop data lifecycle management framework—the system integrates the Data Standardization Loop (input control), Adversarial Triggering Alert Loop (real-time monitoring), and AI Intelligent Verification Loop (semantic feedback). This coupling ensures that clinical data quality is not merely checked post-hoc, but is continuously refined and validated through the synergistic interaction of these three functional stages (35)—a layered and collaborative architecture was designed. This structure consists of four tiers from top to bottom: the Data Layer, Platform Layer, Algorithm Layer, and Application Layer, all deeply interconnected through coordinated data flow, control signals, and feedback mechanisms (36, 37) (Figure 1).

The Data Layer is responsible for ingesting multi-source heterogeneous data. It consolidates 12 categories of information, including EMR, tongue and pulse imaging, laboratory tests, and rehabilitation follow-ups, utilizing Hadoop distributed storage for integration **(**38, 39). The Platform Layer employs a federated learning framework to enable cross-institutional collaborative model training while preserving data privacy. This layer also integrates terminology services and API gateways to ensure system interoperability. Serving as the core, the Algorithm Layer incorporates an adversarially optimized three-tier trigger engine and a domain-adaptive AI model enhanced with bilinear attention mechanisms. Finally, the Application Layer translates these underlying capabilities into specific functions—such as clinical quality control, decision support, and patient management—via endpoints including physician workstations, patient mobile applications, and management dashboards, thereby forming a fully integrated closed-loop system (38, 39).

### 2.2 Construction of the standardized data element system

#### 2.2.1 Design process and principles

**Figure 2:**
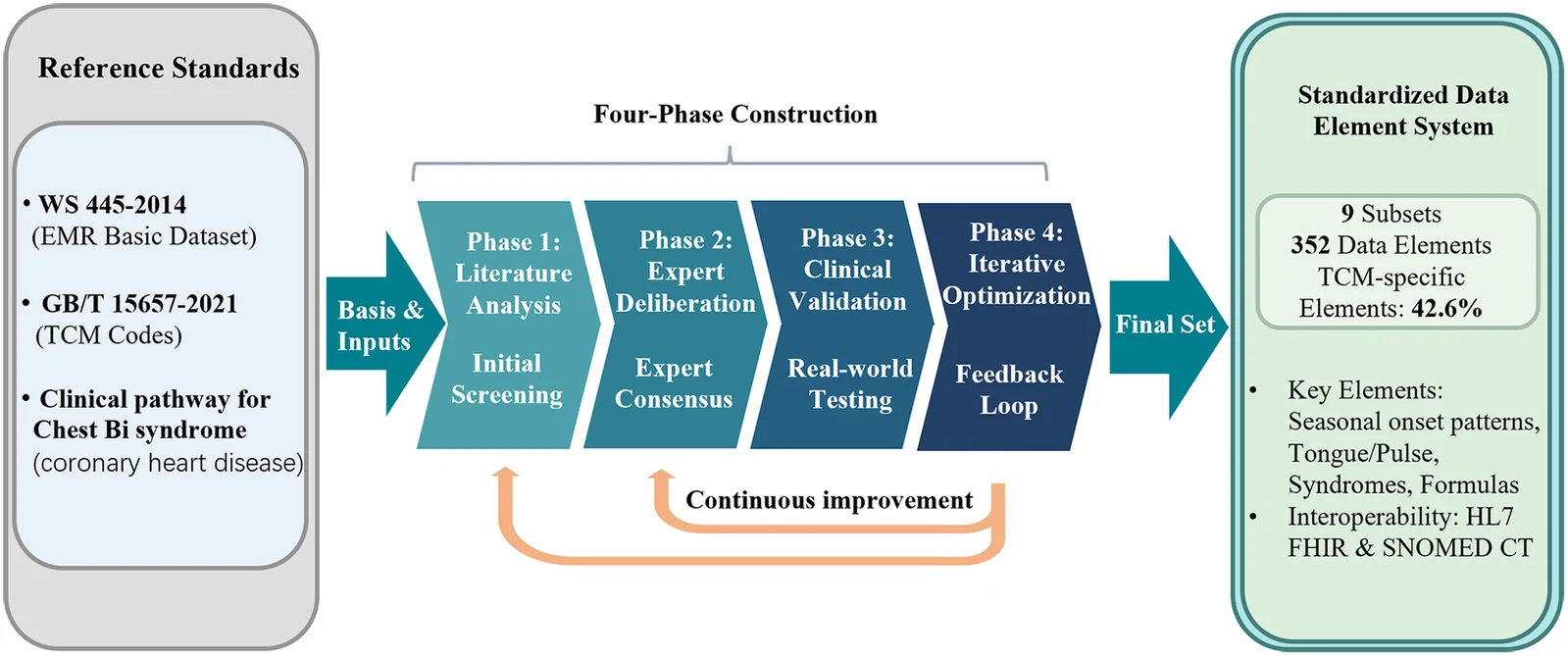
Construction process of the standardized data element system for chest impediment. The system was developed using a four-phase methodology: (1) Literature Analysis for initial screening based on national standards and clinical pathways; (2) Expert Consultation conducted by a multidisciplinary expert panel to refine data elements; (3) Clinical Validation via real-world testing in EMR systems; and (4) Iterative Optimization through a continuous feedback loop. The resulting system comprises 352 data elements categorized into 9 subsets, with 42.6% being TCM-specific items.

A standardized data element system was constructed by adhering to a four-step methodology: “literature analysis → expert deliberation → clinical validation → iterative optimization” (40). This process integrated established national standards—including the WS 445-2014 Basic Dataset for EMR (24) and GB/T 15657-2021 Classification and Coding of TCM Syndromes—alongside the clinical pathway for Chest Bi syndrome (coronary heart disease) (41) (Figure 2). The design was guided by the following key principles: achieving full-process coverage (spanning pre-diagnosis, diagnosis, and post-diagnosis phases); emphasizing TCM characteristics by prioritizing core elements such as the Four Diagnostic Methods, syndrome differentiation, and herbal formula composition; ensuring strong interoperability through compatibility with international standards like HL7 FHIR and SNOMED CT (42, 43); and prioritizing practicality by refining data definitions and value sets based on actual clinical workflows (44, 45).

#### 2.2.2 Core content and terminology mapping

**Figure 3:**
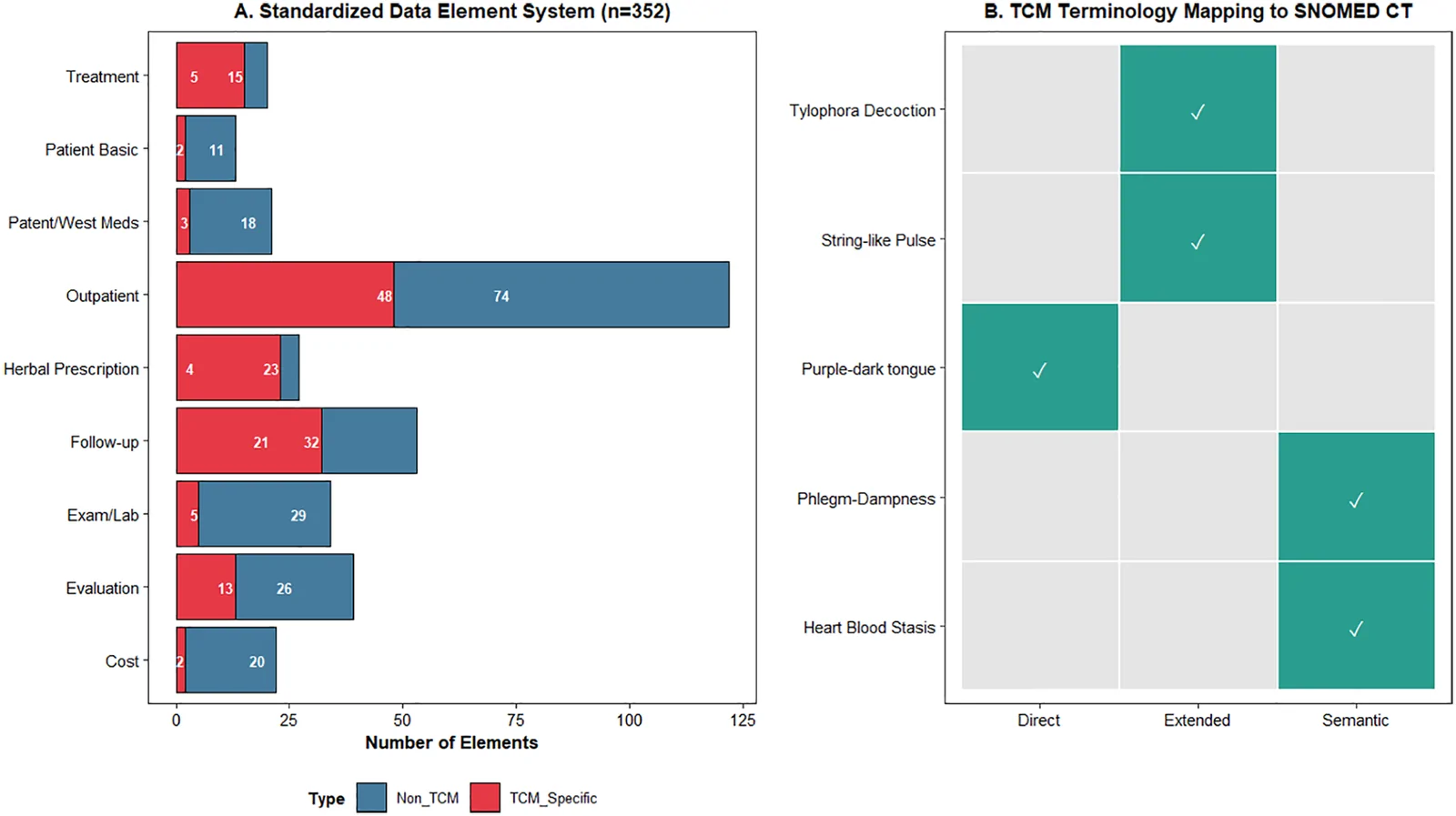
Standardization framework of TCM clinical data.

The finalized system comprises 9 subsets and 352 data elements, which include 150 TCM-specific elements (42.6%). These cover core TCM components such as seasonal onset patterns, tongue quality, pulse conditions, and syndrome codes (Figure 3 Standardization Framework of TCM Clinical Data). Using HL7 FHIR R4, all data elements were mapped to corresponding FHIR resources. Simultaneously, terminology mapping between TCM concepts and SNOMED CT was established, completing the mapping of 137 core TCM terms, achieving a coverage rate of 91.2%. For TCM-specific terminology, standardization was accomplished by extending relevant semantic concepts within SNOMED CT (Figure 3 Standardization Framework of TCM Clinical Data).

**Figure 4:**
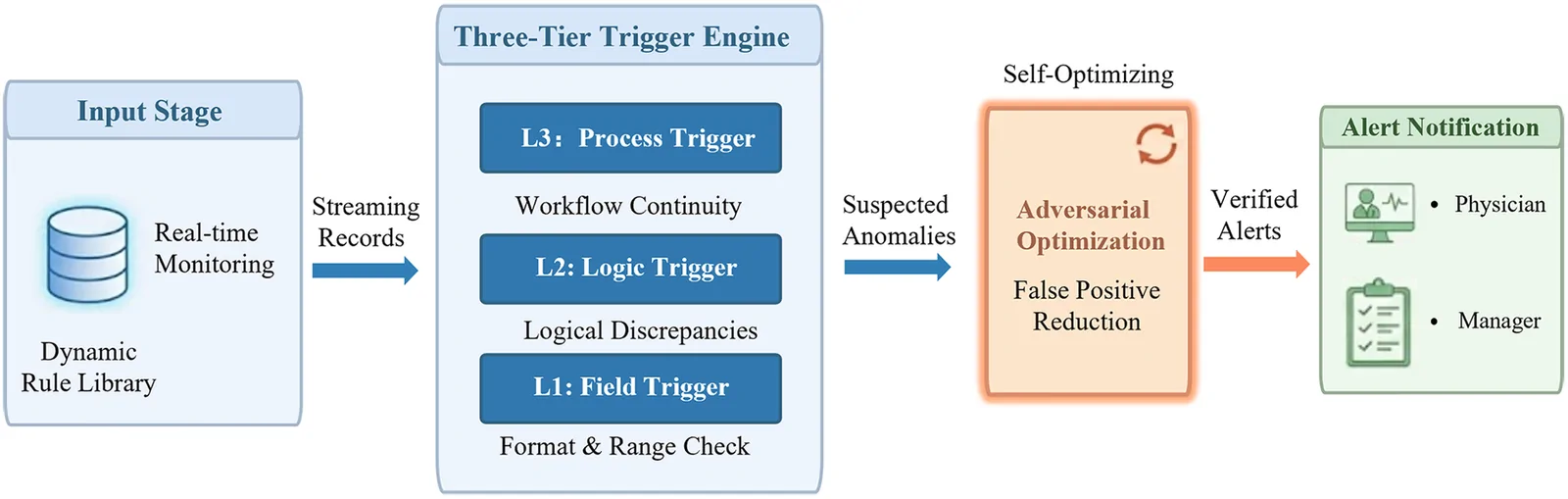
Workflow of the adversarial optimization three-level trigger engine. The process initiates with Real-time Monitoring of streaming clinical records against a Dynamic Rule Library. The engine applies a hierarchical validation mechanism: Level 1 (Field Trigger) checks for format and value range validity; Level 2 (Logic Trigger) ensures logical consistency across fields; and Level 3 (Process Trigger) monitors the continuity of the "prevention–treatment–rehabilitation" workflow. Suspected Anomalies identified by the rules are processed by the Adversarial Optimization Module, which employs self-optimizing algorithms for False Positive Reduction. Only Verified Alerts are propagated to physicians and managers to ensure intervention efficiency.

We established a standardized data element system comprising 9 subsets and 352 data elements. Of these, 150 (42.6%) are TCM-specific. The subsets and their element counts are: Patient Basic Information (13 total, 2 TCM-specific), Outpatient/Emergency Medical Record (122, 48), Chinese Herbal Prescription (27, 23), Chinese Patent Medicine/Western Medicine Prescription (21, 3), General Treatment Procedure Record (20, 15), Examination and Laboratory Test Record (34, 5), Diagnosis and Treatment Cost Information (22, 2), Post-treatment Follow-up Information (53, 32), and Post-treatment Evaluation Information (39, 13). Using HL7 FHIR R4, all data elements were mapped to corresponding FHIR resources. Terminology mapping between TCM concepts and SNOMED CT was performed, achieving 91.2% coverage (46) (137 out of 150 TCM terms successfully mapped). A conceptual overview of the data element system is presented in Figure 2 Construction process of the standardized data element system for Chest Impediment. (B).

### 2.3 Design of a three-level trigger engine for counter-optimization

#### 2.3.1 Working principle

The trigger engine functions as an adversarially optimized, self-correcting filter designed to bridge the gap between rigid rule-matching and complex clinical logic. Beyond traditional static rules, it operates on a workflow of “rule definition → data monitoring → anomaly determination → adversarial filtering → alert notification”. By implementing a “min-max game” between a modality classifier and a rule optimizer, the engine dynamically suppresses false positives, effectively reducing ‘alarm fatigue' (47)—a common failure in traditional quality control systems where physicians are overwhelmed by irrelevant alerts (48). This adversarial process serves to reduce the false positive rate (28, 49) and enhance the accuracy of anomaly detection (50, 51). The overall workflow of the adversarial optimization three-level trigger engine is shown in Figure 4. The initial pool of 128 rules was back-tested against a historical dataset of 1,000 records to establish baseline sensitivity before prospective deployment.

#### 2.3.2 Three-tier trigger design

##### 2.3.2.1 Tier 1 triggers (field level)

Monitor the compliance of individual data elements against 63 predefined rules, including non-null checks, data format validation, and value range verification. Representative examples include: nullity validation for the “Pulse Pattern Code” field; format verification for “Date of Visit” (requiring YYYY-MM-DD); and range checks for “Herbal Medicine Dosage” (0–500 g). Once triggered, the system immediately prompts the operator to supplement or correct the data directly at the input interface, with a response time of less than 1 s (52–54).

##### 2.3.2.2 Tier 2 triggers (logic level)

This tier performs cross-verification based on combined logic across multiple fields through 41 predefined rules, forming the core of the quality control process. Rules are structured in a “condition–action” format. For example:

###### 2.3.2.2.1 Condition

The chief complaint includes “chest pain” or “chest tightness,” and the TCM diagnosis code is not ZFB011 (Heart Blood Stasis Syndrome) or ZFB012 (Phlegm-Dampness Obstruction Syndrome).

###### 2.3.2.2.2 Action

Trigger a “Chief Complaint–Diagnosis Inconsistency” alert. This logic aligns with validated AI-based TCM diagnostic frameworks (55)

###### 2.3.2.2.3 Condition

The syndrome differentiation is “Cold Aggregation in the Heart Vessel,” and the prescription contains cold-natured herbs. Trigger a “Formula–Syndrome Incompatibility” alert. Such logic-level cross-verification is essential for ensuring the precision of TCM pattern classification (55, 56).

###### 2.3.2.2.4 Action

Trigger a “Formula–Syndrome Incompatibility” alert.

Upon triggering, alerts are pushed directly to the physician's workstation and must be acknowledged or addressed within 24 h.

##### 2.3.2.3 Tier 3 triggers (process level)

These monitors ensure data consistency across the entire “Prevention–Treatment–Rehabilitation” continuum through 24 process-oriented rules. Example scenarios include:

- A patient fails to complete follow-up records within 14 days after an outpatient visit.
- Identified constitution type contradicts the syndrome diagnosis in clinical logic.

When activated, the system simultaneously notifies both the attending physician and the case manager to initiate coordinated intervention.

#### 2.3.3 Adversarial optimization mechanism

Inspired by Generative Adversarial Network (GAN) concepts (50), we constructed a modality classifier and a rule optimizer. The modality classifier, implemented as a three-layer feedforward neural network, is designed to discriminate between different types of data anomalies (genuine anomalies vs. false anomalies) and outputs corresponding probability distributions. The rule optimizer adaptively adjusts trigger rule weights through an adversarial loss function, effectively reducing false positive rates (51, 57).

The adversarial loss function is defined as:

$$\begin{array}{cc} L_{adv}(\theta_{T},\;\theta_{V},\;\theta_{D}) & =-\frac{1}{n+k}\overset{n+k}{\underset{i=1}{\sum }}[\tau_{i}logD(o_{i};\;\;\theta_{D})\\ & +(1-\tau_{i})log(1-D(o_{i};\;\;\theta_{D}))] \end{array}$$

Here, $o_{i}$ represents a data sample identified as anomalous, $\tau_{i}$ denotes its true label (1 for genuine anomaly, 0 for false positive), and, $D(o_{i};\theta_{D})$ represents the probability output from the modality classifier.

The modality classifier was implemented as a three-layer feedforward neural network with 256, 128, and 64 neurons in its hidden layers, respectively. The network employed ReLU activation functions and dropout regularization (dropout rate = 0.5) to prevent overfitting. The rule optimizer adaptively adjusted trigger rule weights using a stochastic gradient descent (SGD) optimizer with a learning rate of 0.001. The adversarial training process consisted of 200 epochs, in which the rule optimizer and the modality classifier were trained alternately. Convergence was defined as no decrease in validation loss exceeding 0.1% for 10 consecutive epochs.

### 2.4 Domain-Adaptive AI model enhanced with bilinear attention

#### 2.4.1 Model architecture and training data

The model is built upon the Chinese-BERT-wwm base model (58–60), which features an input dimension of 768, 12 hidden layers, and 12 attention heads. The training corpus integrates multi-source TCM knowledge, comprising:

- 12,000 annotated, structured medical records of Chest Bi syndrome (coronary heart disease);
- Eight classical TCM texts, including Shanghan Lun and Jingui Yaolue;
- Fifteen TCM clinical practice guidelines.

To ensure the robustness of validation, the total dataset was partitioned into training, validation, and test sets in an 8:1:1 ratio. Crucially, this split was performed at the patient level rather than the record level to prevent data leakage, ensuring that no individual patient's data appeared in more than one subset.

#### 2.4.2 Bilinear attention mechanism

A bilinear attention layer is introduced to effectively capture the fine-grained interactions between TCM entities and contextual textual features (30, 31). The attention mechanism is formulated as follows:

$$A=\mathrm{softmax}((1n\cdot {\mathrm{gp}}^{T})\circ G_{T}^{T}W_{t}W_{v}^{T}G_{V})$$

where:

- $G_{T}\;$denotes the contextual text features,
- $G_{V}$represents the TCM entity features,
- $W_{t}$、$W_{v}$ are learnable parameter matrices,
- ∘ indicates the Hadamard (element-wise) product,
- 1 is an all-ones vector, and p is a trainable pooling parameter vector (61, 62)

#### 2.4.3 Two-stage training strategy

The model was developed using a two-stage training strategy. In the domain pre-training phase (63, 64), it was trained on a specialized TCM text corpus using the masked language modeling (MLM) task (53) (with a learning rate of 5e-5 for 10 epochs) to assimilate the linguistic patterns and domain-specific knowledge of traditional Chinese medicine. In the subsequent task-specific fine-tuning phase, the model was adapted for three downstream tasks (65)—entity extraction, semantic validation, and recommendation generation—via fine-tuning (using a cross-entropy loss function, a learning rate of 3e-5, and trained for 20 epochs). To mitigate overfitting, gradient clipping (threshold: 1.0) and early stopping (patience = 5) strategies were implemented throughout the training process.

The entire dataset was partitioned into training, validation, and test sets in an 8:1:1 ratio using stratified random sampling.

### 2.5 Study design and evaluation methods

#### 2.5.1 Study population

Inclusion criteria: ① age ≥ 18 years; ② primary diagnosis of Chest Bi syndrome (coronary heart disease) (ICD-10: I20–I25); ③ hospitalization between January 1, 2025 and September 30, 2025; ④ complete clinical data with available baseline and follow-up information.

Exclusion criteria: ① severe hepatic or renal insufficiency, malignant tumor, or psychiatric disorders; ② refusal to participate or loss to follow-up; ③ death during hospitalization.

A total of 972 patients (age ≥18 years) diagnosed with Chest Bi syndrome (coronary heart disease) (ICD-10: I20–I25) at Zhongshan Hospital of Traditional Chinese Medicine between January and September 2025 were enrolled. Exclusion criteria included severe hepatic or renal insufficiency, psychiatric disorders, cognitive impairment, and unwillingness to participate in the data collection process.

To ensure the accuracy and consistency of data annotation, a rigorous quality control process was established. For key unstructured text data such as TCM syndrome patterns, tongue, and pulse manifestations, independent double-blind annotation was performed by two associate chief TCM physicians with over 10 years of clinical experience. Inter-annotator agreement was assessed using Cohen's Kappa coefficient (Kappa = 0.85). For records with discrepant annotations, a third, more senior chief physician arbitrated to establish the final gold standard, thereby ensuring data quality for subsequent AI model training and evaluation.

#### 2.5.2 Grouping and evaluation indicators

This study used a non-randomized, parallel-group controlled design. This design was chosen primarily to avoid contamination between the two interventions within the same clinical setting, as the new intelligent system was implemented as the standard of care in designated hospital wards. Patients admitted to the designated “intelligent system wards” between January and September 2025 were consecutively assigned to the intervention group (*n* = 486). Concurrently, patients admitted to comparable “conventional wards” during the same period, who received the traditional manual quality control process, formed the control group (*n* = 486). Baseline characteristics between the two groups were well-balanced (all *P* > 0.05), supporting comparability.

Evaluation metrics included:

- Data Quality: Core data element collection rate, TCM-specific data elements element collection rate, data accuracy, logical consistency error rate, and unstructured data conversion rate (63, 66);
- Quality Control Efficiency: Time required for reviewing each medical record, daily processing capacity, and alert response time;
- Model Performance: Precision, recall, and F1-score of the AI model, along with accuracy in detecting anomalies via triggers (64);
- Clinical Acceptance: Physician satisfaction evaluated using a 5-point Likert scale (44).

#### 2.5.3 Statistical analysis

All statistical analyses were performed using R version 4.5.1. Continuous variables are expressed as mean ± standard deviation (x ± s) and were compared using independent samples t-test. Categorical variables are presented as frequencies and percentages (n, %) and were compared using the chi-square (*χ*^2^) test. To control for Type I error inflation due to multiple comparisons, the Benjamini-Hochberg false discovery rate (FDR) method was applied to adjust *p*-values across all primary outcomes (67, 68) (data completeness, TCM-specific data elements collection rate, logical consistency error rate, etc.). An adjusted *p*-value (P_adj) < 0.05 was considered statistically significant. Effect sizes were calculated to quantify the magnitude of differences: Cohen's d for continuous variables and Cramér's V for categorical variables. For key comparisons, 95% confidence intervals (CIs) were also reported. Repeated measures were analyzed where appropriate (33), and internal auditing principles were followed for quality validation (34).

#### 2.5.4 Ethics statement

This study was approved by the Institutional Review Board of Zhongshan Hospital of Traditional Chinese Medicine [Approval No. KY (2024) No. 086-106, date of approval: October 11, 2024]. This was a non-interventional, observational study that analyzed de-identified data from routine clinical care to evaluate a novel data governance system. The IRB waived the requirement for informed consent because the study posed no more than minimal risk to patients and involved no direct patient intervention. All patient data were anonymized before analysis to ensure privacy protection.

## 3 Results

A total of 972 patients diagnosed with Chest Bi syndrome (coronary heart disease) were enrolled in this prospective controlled study, with 486 patients assigned to the intervention group (intelligent data governance and quality control system) and 486 to the control group (conventional manual quality control). The primary outcomes included multidimensional data quality, quality control efficiency, performance of the three-tier trigger engine and domain-adaptive AI model, ablation effects of system modules, parameter sensitivity, long-term effectiveness, and clinical acceptability. All statistical comparisons were adjusted for multiple testing using the Benjamini–Hochberg FDR method, with adjusted P (P_adj) < 0.05 considered statistically significant.

### 3.1 Baseline characteristics were well balanced between groups

**Figure 5:**
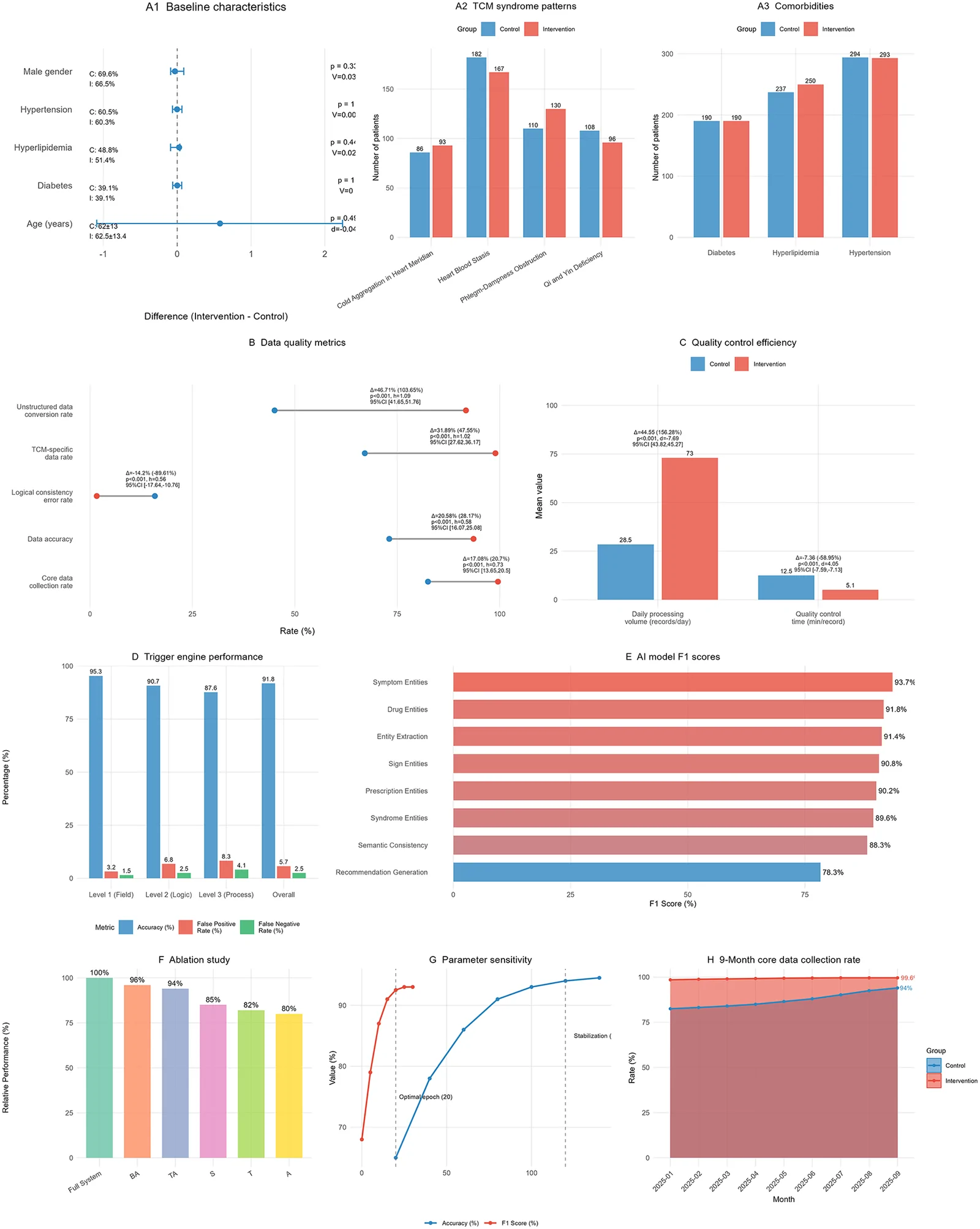
Overall performance of the intelligent data governance and quality control system for chest Bi syndrome (coronary heart disease). (A1–A3) Baseline characteristics were comparable between groups (all *P* > 0.05). **(B)** Data quality was significantly improved across five core indicators (all P_adj < 0.001). **(C)** Quality control efficiency was markedly enhanced in the intervention group. **(D)** High accuracy and low false positive/negative rates of the three-tier trigger engine. **(E)** Strong performance of the bilinear attention–enhanced domain-adaptive AI model. **(F)** Ablation study showing synergistic effects of the three core modules. **(G)** Parameter stability of trigger rules and AI training epochs. **(H)** Sustained data quality and higher physician satisfaction over 9 months.

Baseline demographic and clinical characteristics were comparable between the intervention and control groups, with no statistically significant differences detected (all *P* > 0.05), ensuring valid comparative analysis (Figures 5A1–A3). The mean age was 62.53 ± 13.39 years in the intervention group and 61.95 ± 13.03 years in the control group [t = −0.68, *P* = 0.498; 95% confidence interval (CI) −2.24 to 1.09; Cohen's d = −0.043]. The proportion of male patients was 66.46% vs. 69.55% (*χ*^2^ = 1.06, *P* = 0.336; Cramér's V = 0.033; 95% CI −2.8% to 8.9%). Mean disease duration was 5.37 ± 2.96 vs. 5.43 ± 2.96 years (t = 0.32, *P* = 0.750; 95% CI −0.31 to 0.43; Cohen's d = 0.020). Distributions of TCM syndrome patterns, including Heart Blood Stasis, Phlegm-Dampness Obstruction, Cold Aggregation in Heart Meridian, and Qi and Yin Deficiency, were similar between groups (*χ*^2^ = 3.29, *P* = 0.349). The prevalence of major comorbidities, including hypertension (60.3% vs. 60.5%), diabetes (39.1% vs. 39.1%), and hyperlipidemia (51.4% vs. 48.8%), also showed no intergroup differences (all *P* > 0.05).

### 3.2 Intelligent governance significantly improved multidimensional data quality

The intelligent system yielded substantial and statistically significant improvements across all five core data quality indicators in the intervention group compared with the control group (P_adj < 0.001; Figure 5B).

Core data completeness increased from 82.51 ± 38.03% to 99.59 ± 6.41%, with an absolute improvement of 17.08% (95% CI 13.65 to 20.50; Cohen's h = 0.73).

TCM-specific data elements collection rate rose from 67.08 ± 47.04% to 98.97 ± 10.10%, with an absolute improvement of 31.89% (95% CI 27.62 to 36.17; Cohen's h = 1.02).

Overall data accuracy improved from 73.05 ± 44.42% to 93.62 ± 24.46%, with an absolute improvement of 20.58% (95% CI 16.07 to 25.08; Cohen's h = 0.58).

Logical consistency error rate decreased from 15.84 ± 36.55% to 1.65 ± 12.74%, with an absolute reduction of 14.20% (95% CI −17.64 to −10.76; Cohen's h = 0.56).

Unstructured data conversion rate increased from 45.06 ± 49.81% to 91.77 ± 27.51%, with an absolute improvement of 46.71% (95% CI 41.65 to 51.76; Cohen's h = 1.09).

These improvements demonstrated that the standardized framework combined with real-time triggering and AI verification effectively enhanced the completeness, accuracy, consistency, and structuralization of clinical data, especially for TCM-specific information.

### 3.3 Quality control efficiency was markedly enhanced

Building upon the improvements in data quality, the system's impact on clinical workflow efficiency was evaluated. Quality control efficiency was significantly improved in the intervention group, reflecting strong practical value for clinical workflow (Figure 5C). The mean time required for quality control per medical record was reduced from 12.48 ± 2.31 min in the control group to 5.12 ± 1.14 min in the intervention group, representing an absolute reduction of 7.36 min (95% CI −7.59 to −7.13; *P* < 0.001; Cohen's d = 4.05) and a relative efficiency improvement of 58.95%. The daily processing capacity of quality control personnel increased from 28.50 ± 4.38 records to 73.05 ± 6.92 records, with an absolute increase of 44.55 records (95% CI 43.82 to 45.27; *P* < 0.001; Cohen's d = −7.69). Among the intervention group, the median alert response time was 2.3 min [interquartile range (IQR) 1.6 to 3.2 min], supporting rapid correction of data anomalies.

### 3.4 The three-tier adversarial trigger engine achieved high detection accuracy

To identify the mechanisms driving these quality gains, we specifically analyzed the performance of the three-tier adversarial trigger engine. The adversarially optimized three-tier trigger engine demonstrated reliable performance in real-time data anomaly detection (Figure 5D). Stratified by trigger levels:

Level 1 (field-level format and range validation): 95.3% accuracy, 3.2% false positive rate, and 1.5% false negative rate.
Level 2 (logic-level cross-field consistency verification): 90.7% accuracy, 6.8% false positive rate, and 2.5% false negative rate.
Level 3 (process-level prevention–treatment–rehabilitation continuity monitoring): 87.6% accuracy, 8.3% false positive rate, and 4.1% false negative rate.

The overall performance of the trigger engine reached 91.8% accuracy, with a 5.7% false positive rate and 2.5% false negative rate. Adversarial learning optimization effectively reduced the false positive rate by 4.2% compared with conventional rule-based triggers, markedly improving the precision and clinical usability of alerts.

### 3.5 Domain-adaptive AI with bilinear attention showed strong NLP performance

The domain-adaptive AI model based on Chinese-BERT-wwm and enhanced with a bilinear attention mechanism achieved excellent performance in TCM clinical text processing (Figure 5E). The overall F1-score for named entity extraction from unstructured medical records was 91.4%. Stratified by entity type:

Symptom entities: 93.7% F1-score
Sign entities: 90.8% F1-score
Drug entities: 91.8% F1-score
Syndrome entities: 89.6% F1-score
Prescription entities: 90.2% F1-score

For semantic consistency verification of TCM diagnostic and therapeutic information, the model achieved an F1-score of 88.3%. The clinical adoption rate of intelligent correction and recommendation suggestions reached 78.3%. In a benchmarking analysis, the integrated domain-adaptive model outperformed the vanilla baseline in overall F1-score, with the bilinear attention mechanism specifically contributing a 3.1% improvement.

### 3.6 Ablation studies and parameter sensitivity validate system robustness

Ablation experiments confirmed the synergistic contributions of the three core modules (standardization framework, adversarial trigger engine, and domain-adaptive AI) to overall system performance (Figure 5F). The complete integrated system was defined as 100% relative performance. Removing the standardization framework reduced performance to 85%, removing the trigger engine reduced performance to 82%, and removing the AI module reduced performance to 80%. The adversarial trigger engine alone achieved 94% relative performance, and the bilinear attention module alone achieved 96% relative performance.

Parameter sensitivity analysis revealed clear optimal operating ranges (Figure 5G). System detection accuracy increased with the number of trigger rules and stabilized at 94.0%–94.5% when the number of rules exceeded 120. The F1-score of the AI model increased with fine-tuning epochs, peaked at approximately 92.5% at 20 epochs, and plateaued thereafter, indicating adequate convergence without overfitting. These results validate the synergistic effect of the Triple-Loop Coupling framework, where the absence of any single loop leads to a significant degradation in the system's ability to maintain data integrity.

### 3.7 Long-term effectiveness and high clinical acceptability

Over the 9-month study period (January to September 2025), the intervention group maintained stable and high-level data quality, demonstrating the robustness of the intelligent system (Figure 5H). The core data collection rate remained consistently ≥98%, rising from 98.5% in January to 99.6% in September. In contrast, the control group showed only gradual improvement from 82.5% to 94.0% over the same period. Clinical acceptability was significantly higher in the intervention group, with a mean physician satisfaction score of 4.3 ± 0.6 compared with 2.7 ± 0.8 in the control group (*P* < 0.001). These results confirmed the long-term stability, effectiveness, and clinical usability of the intelligent data governance and quality control system.

## 4 Discussion

The primary innovation of the intelligent data governance system developed in this study lies in its integration of a Triple-Loop Coupling—"standardization, adversarial triggering (70), and AI verification"—systematically addressing the core challenges in TCM clinical data governance. Compared with existing approaches, the system demonstrates three distinct advantages:

First, the standardized system emphasizes both TCM specificity and interoperability. Among the 352 data elements established, TCM-specific elements constitute 42.6%, significantly higher than proportions reported in comparable studies. Through systematic mapping to HL7 FHIR (6) and SNOMED CT, the system enables cross-system integration of multi-source data, effectively breaking down data silos and establishing a robust foundation for data quality enhancement. This is clearly reflected in the results: data completeness improved from 82.51% to 99.59%, while the collection rate of TCM-specific elements increased from 67.08% to 98.97%, demonstrating the substantial effectiveness of the standardized framework (42, 43).

Second, the adversarially optimized trigger mechanism effectively overcomes limitations inherent in traditional rule-based systems (57). The implementation of this adversarial engine addresses a critical bottleneck identified in system-level AI research: the delicate balance between monitoring sensitivity and clinical alarm fatigue (14). With the incorporation of adversarial learning, the trigger engine achieved 91.8% accuracy in anomaly detection, while the false positive rate dropped to 5.7%—a 4.2% reduction compared to conventional triggers. By dynamically filtering irrelevant alerts, the system aligns with the broader paradigm shift towards precision medicine (64, 69) and global efforts to improve the robustness and clinical usability of decision support tools. This real-time alerting capability eliminates the inherent delay of post-event quality control, reducing the time required for single-record review by 58.95% and substantially enhancing operational efficiency (70).

Third, the integration of bilinear attention mechanisms significantly enhances the AI model's adaptability to TCM-specific contexts. Through domain-specific pre-training on TCM corpora and the implementation of bilinear attention, the model achieves a 91.4% F1-score in TCM entity recognition (74, 75), while substantially elevating the conversion rate of unstructured clinical text. The bilinear attention mechanism contributes an additional 3.1% performance gain. These results collectively demonstrate the model's enhanced capacity for precise semantic understanding and information extraction from specialized TCM text, consistent with broader findings on the utility of AI in managing complex clinical information (73).

This study has several limitations. First, as the system was developed and validated in a single medical center using Chest Bi syndrome as a demonstration disease, the findings should be interpreted as a preliminary exploratory success. Consequently, conclusions regarding the system's scalability and generalizability should be viewed as preliminary. Extrapolation to other TCM specialties or disease domains requires caution and awaits validation through future multi-center studies.Future multi-center studies are essential to establish the external validity and generalizability of the Triple-Loop Coupling framework across diverse institutional settings. Second, the AI model's semantic understanding of rare TCM syndromes and complex herbal formulations remains suboptimal and requires further enhancement. Third, the maintenance of trigger rules still relies on manual expert input. Fourth, this study primarily focused on quality and efficiency metrics of the data governance process itself. Although high-quality clinical data is a crucial foundation for improving patient outcomes, this study did not directly evaluate the system's impact on long-term clinical endpoints. Making this a key direction for future research will help more comprehensively validate the system's value in enhancing real-world clinical effectiveness.Future research will focus on: (1) conducting multi-center studies to verify the system's generalizability across diverse clinical settings; (2) implementing federated adversarial learning approaches to address data privacy constraints while improving model robustness; (3) integrating TCM knowledge graphs to strengthen the AI model's capability in theoretical reasoning; and (4) expanding applications to additional TCM-advantaged diseases such as stroke and diabetes (74).

This study has several limitations. First, the system was developed and validated in a single medical center using Chest Bi syndrome as a demonstration disease.Second, the AI model's semantic understanding of rare TCM syndromes remains suboptimal.Third, the maintenance of trigger rules still relies on manual expert input.

Furthermore, the control group in this study underwent weekly random sampling at a specified proportion for quality control, which reflects the real-world practice in our center before the intelligent system was deployed. However, this creates a methodological asymmetry because the intervention group was monitored in real time. Although baseline characteristics were well balanced, the difference in monitoring intensity may favor the intervention group beyond the technical merits of the system. Future studies should adopt concurrent real-time monitoring in both arms to eliminate this asymmetry.

We did not perform a formal cost-effectiveness analysis. The reported efficiency gains, including a 58.95% reduction in quality control time per record — suggest substantial potential for cost savings. However, direct costs such as hardware, software development, and staff training were not quantified. Therefore, the economic viability of implementing this system in other institutions remains to be determined. Future studies should include a full health economic evaluation (e.g., cost-benefit or return-on-investment analysis) to support broader adoption.

## 5 Conclusion

This study establishes an intelligent data governance and quality control system that synergistically improves both data quality and control efficiency throughout the entire “prevention–treatment–rehabilitation” continuum for TCM-advantaged diseases. This is achieved through three integrated components: establishing a standardized data framework to build a robust foundation, implementing adversarially optimized triggers for real-time monitoring and alerts, and leveraging domain-adaptive AI to enhance intelligent processing capabilities. The system's technical architecture is designed to be extendable, and it provides a preliminary and verifiable exploratory framework for the standardized governance and intelligent quality control of TCM clinical data. It holds significant potential for broader application in the digital transformation of other TCM-advantaged disease areas, thereby providing robust data support for the modernization and global dissemination of traditional Chinese medicine (27).

## Data Availability

The datasets presented in this article are not readily available due to privacy and ethical restrictions imposed by the Institutional Review Board of Zhongshan Hospital of Traditional Chinese Medicine [Approval KY (2024) No. 086-106]. De-identified data can be made available to qualified researchers upon reasonable request and with written approval from the IRB.Requests should be directed to the corresponding author (80379721@qq.com) and will be responded to within four weeks. Requests to access the datasets should be directed to 80379721@qq.com.
